# Supplementary material for: Magnetopause ripples going against the flow form azimuthally stationary surface waves
Source: Nat Commun. 2021 Oct 6;12:5697. doi: 10.1038/s41467-021-25923-7 (PMC8494893; doi:10.1038/s41467-021-25923-7)
Supplement: Supplementary file 1 — Supplementary information [file 41467_2021_25923_MOESM1_ESM.pdf]

Supplementary information for "Magnetopause ripples going against the flow form azimuthally stationary surface waves"

M. O. Archer, M. D. Hartinger, F. Plaschke, D. J. Southwood, & L. Rastaetter

Simulation Setup		
Dipole GSM Orientation		(0,0,1)
Dipole Update		No
Solar Wind Conditions		
Quantity	Solar Wind	1 min Density Pulse
n (cm^{-3})	6	14
T (K)	116,174.0	49,788.8
$\mathbf{v}_{GSM}$ ($\mathrm{km\,s^{-1}}$)	(-450,0,0)	(-450,0,0)
$\mathbf{B}_{GSM}$ (nT)	(0,0,5)	(0,0,5)
GSM normal		(1,0,0)
Ionospheric Conditions		
Conductivity		5 mho (uniform)

Supplementary Table 1: Details of the SWMF (Space Weather Modeling Framework) global magnetohydrodynamic simulation run used in this paper. The first subgroup details the setup of the magnetic dipole in Geocentric Solar Magnetospheric (GSM) coordinates. The second subgroup lists the plasma number density n, temperature T, velocity $\mathbf{v}$, and magnetic field $\mathbf{B}$ in both the ambient solar wind and the 1 min density pulse, along with the latter's orientation. The final subgroup highlights the ionospheric conditions used.

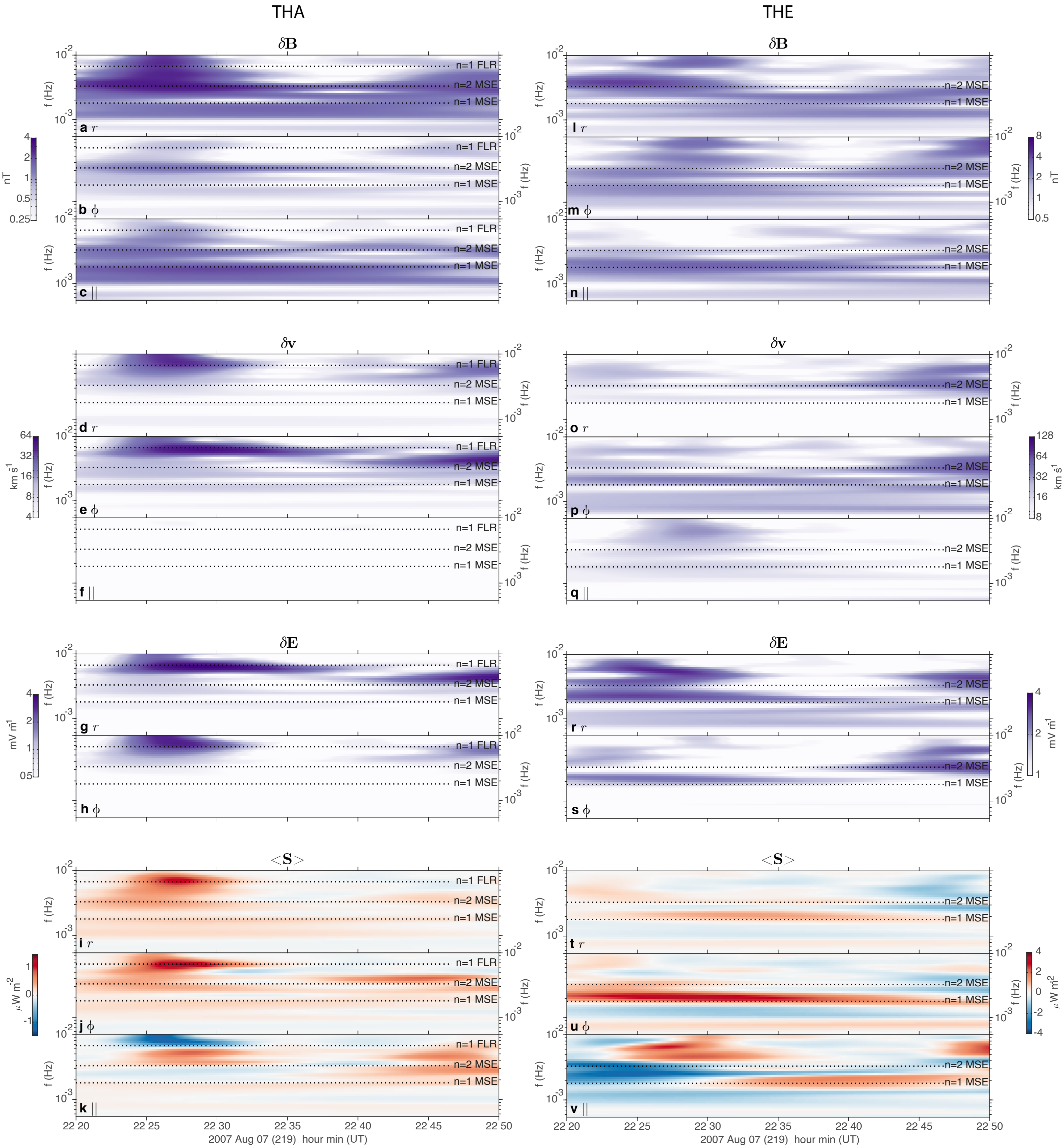

Supplementary Fig. 1: Dynamic spectra of the spacecraft observations using the wavelet transform. Shown for THA (a–k) and THE (l–v). Panels show radial (top), azimuthal (middle), and field-aligned (bottom) components of the magnetic (a–c, l–n), velocity (d–f, o–q), and electric (g–h, r–s) field perturbations, with the logarithmic colour scales indicating amplitudes. The components of the time-averaged Poynting vector as function of frequency and time are depicted in the final panels (i–k, t–v). Dotted lines depict MSE and local FLR frequencies.

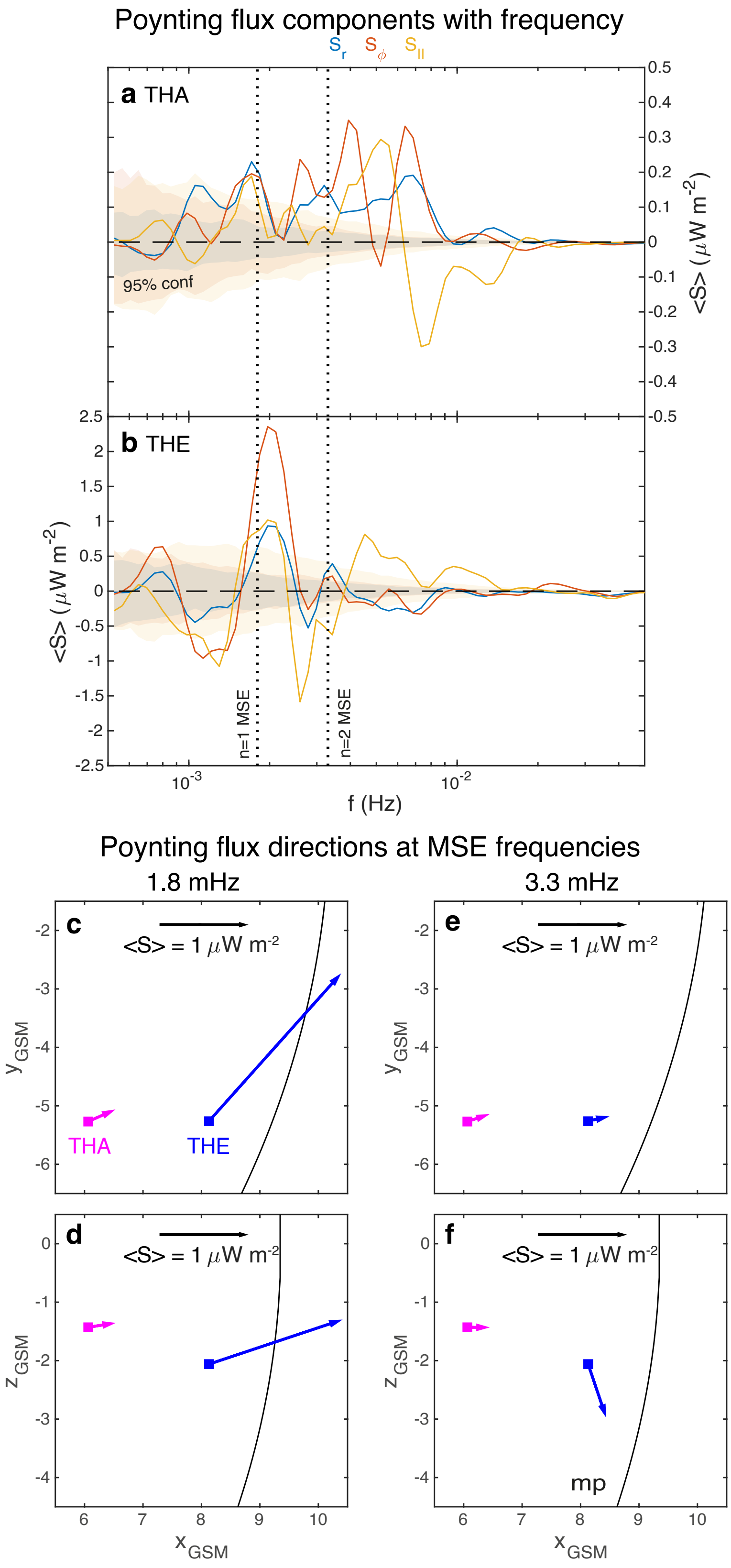

Supplementary Fig. 2: Poynting fluxes averaged over the interval. These are shown by component as a function of frequency for THA (a) and THE (b). Shaded areas indicate 95% confidence intervals for null hypotheses of autoregressive noise for each component. The two MSE frequencies are highlighted by vertical dotted lines. Subsequent panels show, at the two MSE frequencies, the Poynting fluxes in the $z_{GSM} = -2.1\,\mathrm{R_E}$ (c,e) and $y_{GSM} = -5.3\,\mathrm{R_E}$ (d,f) planes as arrows originating from the spacecraft locations (squares). A model magnetopause is also shown (black).

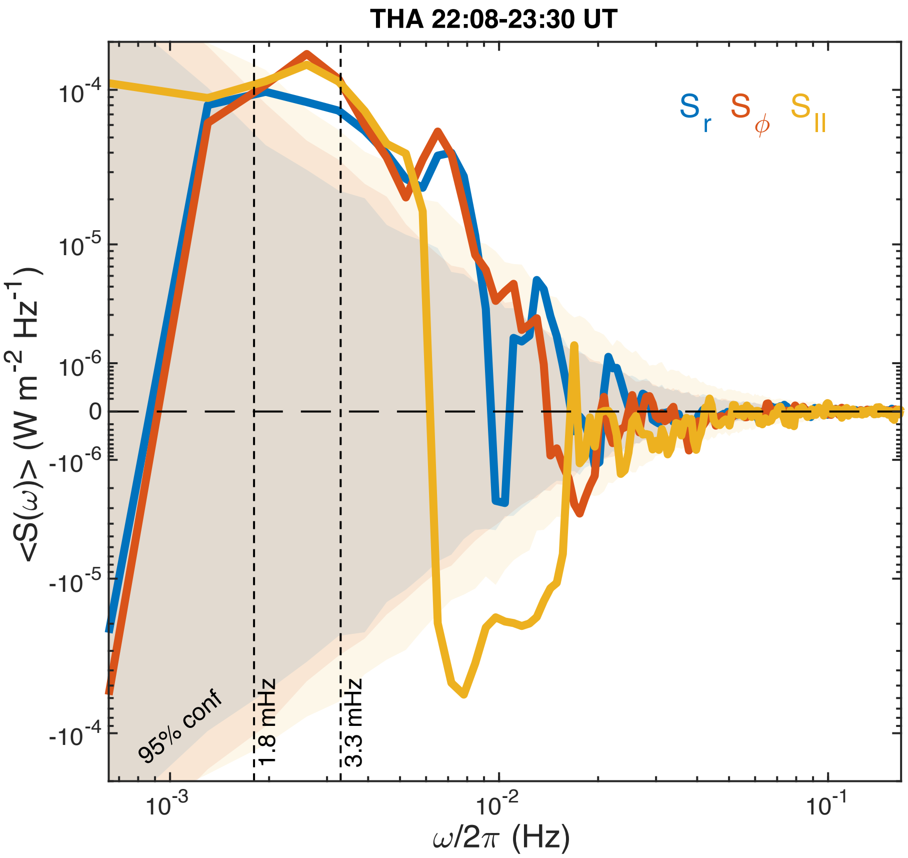

Supplementary Fig. 3: Components of the time-averaged Poynting vector at THA as a function of frequency over an extended time interval. A bi-symmetric log scale is used on the vertical axis. Shaded coloured areasindicate 95% confidence intervals for null hypotheses of autoregressive noise for each component. The identified MSE frequencies are indicated by vertical dotted lines.

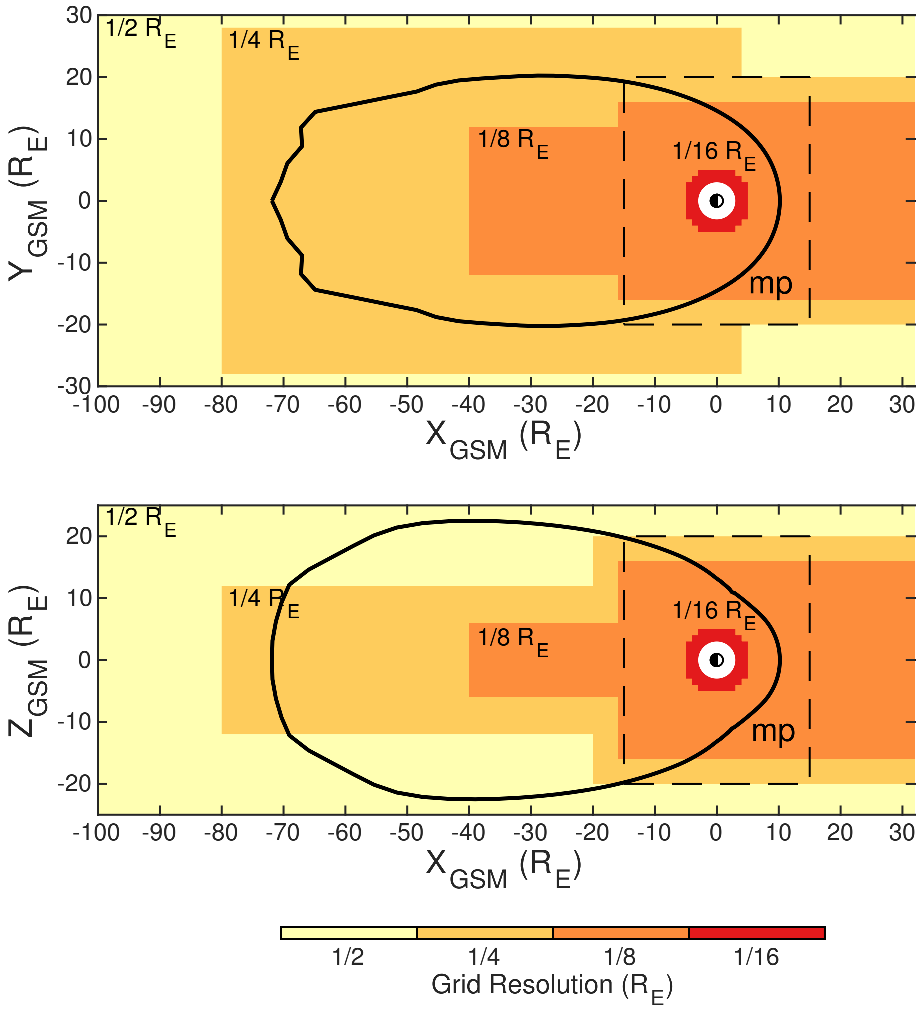

Supplementary Fig. 4: Grid resolution of the global MHD simulation. Shown for the GSM XY (top) and XZ (bottom) planes. The black line depicts the equilibrium boundary of closed field lines, used as a proxy for the magnetopause location in this paper. The dashed box indicates the region of interest in this paper.
