# Supplementary material for: Magnetopause ripples going against the flow form azimuthally stationary surface waves
Source: Nat Commun. 2021 Oct 6;12:5697. doi: 10.1038/s41467-021-25923-7 (PMC8494893; doi:10.1038/s41467-021-25923-7)
Supplement: Supplementary file 3 — Description of Additional Supplementary Files [file 41467_2021_25923_MOESM3_ESM.docx]

File Name: Supplementary Movie 1
Description: Movie of the global MHD simulation run in the GSM XY (left) and XZ (right) planes. Colours depict perturbations in the local field-aligned (compressional) magnetic field component with a bi-symmetric log transform applied. The magnetopause location (black) is also indicated, identified as the last closed field line using a bisection method. This has been checked against field-line topology flags to ensure it reliably provides the boundary between field lines connected to Earth (which may also include tail lobe field lines) and purely solar wind field lines (not connected to Earth at all) and is reliable in the region depicted. Field line tracings are also shown in the XZ plane (grey) and hours of magnetic local time in the XY plane (dotted).

File Name: Supplementary Movie 2
Description: Zoom in of Supplementary Movie 1 highlighting the stationary (right) and propagating (left) regions in the GSM XY plane.
